# Kienböck’s Disease: A Rare Case Report and Review of Management

**DOI:** 10.7759/cureus.82322

**Published:** 2025-04-15

**Authors:** Matthew Thomas, Peter Richa, Jalal Ibrahim, Feross Habib, Robert Savarese

**Affiliations:** 1 Pediatrics and Child Health, Lake Erie College of Osteopathic Medicine, Bradenton, USA; 2 School of Medicine, Lake Erie College of Osteopathic Medicine, Bradenton, USA; 3 Medical School, Lake Erie College of Osteopathic Medicine, Bradenton, USA; 4 Medicine, Lake Erie College of Osteopathic Medicine, Bradenton, USA; 5 Physiatry, Jacksonville Orthopaedic Institute, Jacksonville, USA

**Keywords:** avascular necrosis, kienböck's disease, lichtman classification, lunate avn, lunate osteonecrosis, lunatomalacia

## Abstract

Kienböck’s disease (KD) is a rare condition characterized by avascular necrosis of the lunate, ultimately causing progressive wrist dysfunction and pain. Here, we present a case of a 26-year-old woman with persistent left wrist pain refractory to conservative treatment. Physical examination revealed tenderness over the lunate with diminished sensation in the ulnar and median nerve distribution. However, nerve conduction studies showed normal nerve function, ruling out carpal tunnel syndrome. Various imaging modalities revealed lunate sclerosis, edema, and ulnar negative variance, strongly indicative of KD. In this case, the patient decided to undergo a left distal radius shortening osteotomy to alleviate lunate compression and restore vascular perfusion. This case report highlights the multifactorial nature of KD, including anatomical, vascular, occupational, and genetic risk factors. Additionally, this case emphasizes the importance of tailoring treatment modalities based on disease severity as outlined by the Lichtman classification system. Given the limited literature on KD and the absence of standardized treatment guidelines, further research is necessary to optimize treatment strategies and improve clinical outcomes.

## Introduction

Wrist pain is a common ailment that may arise from various causes, including trauma, overuse, or underlying medical conditions. Among the many potential causes of wrist pain, one often overlooked but significant condition is Kienböck's disease (KD). KD is a rare disorder of the wrist that was documented by Austrian radiologist Robert Kienböck in 1910 [1]. The radiologist first described the disease as a ligamentous injury to the lunate that compromises its internal arterial supply [1]. 

Presently, a combination of vascular, anatomical, and traumatic factors is thought to be at play, although its distinct etiology has yet to be discovered [2]. KD remains a very unique disorder with limited literature about therapeutic recommendations and risk factors. KD primarily affects males between the ages of 20 and 40. If left untreated, it may progress to chronic pain and wrist dysfunction, underscoring the importance of early diagnosis and intervention for a favorable prognosis [2]. This case report is important as it adds to the limited literature on KD, emphasizing the need for early recognition and standardized treatment approaches to enhance patient outcomes. Here, we highlight the management of a 26-year-old woman with KD and explore the various possible etiologies and treatment modalities.

## Case presentation

We present the case of a 26-year-old woman who returned for a follow-up visit in physiatry due to left wrist pain. The patient had previously been seen for wrist pain, but her pain levels showed minimal improvement with conservative treatment. She describes the pain as sharp, throbbing, and stabbing, occurring at rest with intermittent exacerbations of painful episodes. The patient states that the pain is typically a 5 out of 10, with the most severe pain reaching a 9 out of 10. She reports that the pain is affecting her sleep and is related to activity. She denies any recent trauma.

The patient is employed as a medical aesthetician and lives independently. She denies ever using tobacco products and admits to occasionally consuming alcoholic beverages. Her past medical history includes anemia, anxiety disorder, depression, and heart disease. Her surgical history includes anterior cruciate ligament repair, right knee arthroscopy, partial right lateral meniscectomy, and tonsillectomy/adenoidectomy. She takes semaglutide for weight loss, spironolactone for heart disease, and oral contraceptives to reduce heavy menstrual bleeding. Her family history is noncontributory.

The physical examination revealed normal vital signs, with a height of 5 feet 8 inches and a weight of 220 pounds (BMI of 33.57). The patient had intact skin bilaterally, with no swelling or signs of infection. However, on palpation, tenderness was noted over the left carpus, particularly in the central area near the lunate. Pulses were palpable and identified as 2+, with brisk capillary refill bilaterally. Somatosensory testing, including light touch, pinprick, and proprioception, revealed diminished sensation in the left ulnar and median nerve distributions, with no apparent atrophy. Reflexes appeared normal bilaterally. Pain was elicited with wrist extension but not flexion; there was good motion otherwise.

Nerve conduction studies were performed in the Physiatry office to further investigate the cause of the wrist pain. However, the nerve conduction study revealed no evidence of carpal tunnel syndrome, demonstrating normal median nerve function without signs of entrapment.

**Figure 1:**
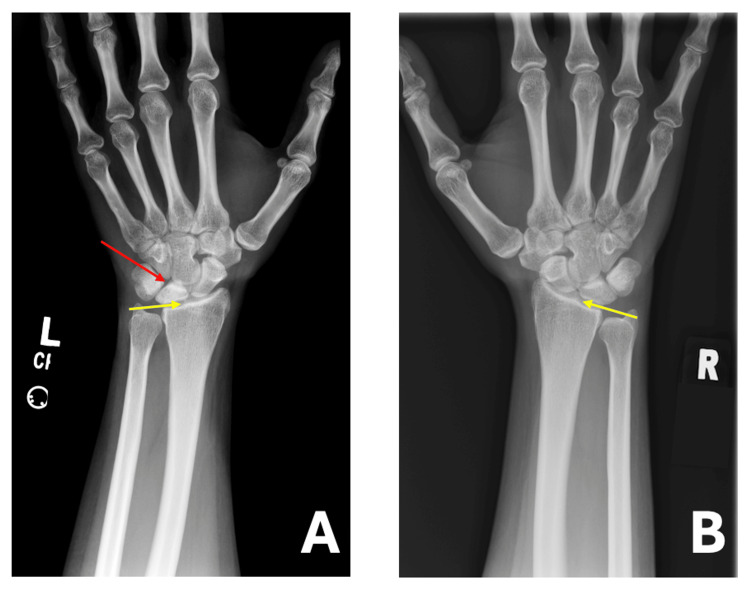
(A, B) Bilateral PA wrist radiographs. (A) Left wrist: The yellow arrow indicates negative ulnar variance, while the red arrow highlights lunate density. (B) Right wrist: The yellow arrow represents negative ulnar variance, with no evidence of lunate alteration. PA: posteroanterior.

**Figure 2:**
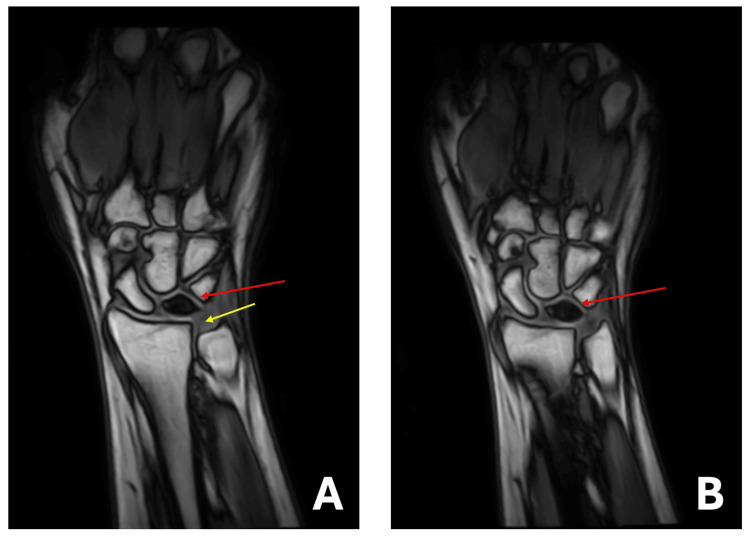
(A, B) Multiplanar multisequence MRI of T1-weighted images of the left wrist. The yellow line indicates negative ulnar variance, while the red lines show decreased signal intensity representative of lunate osteonecrosis. MRI: magnetic resonance imaging.

An X-ray of the left wrist was performed, showing a dense lunate with possible surrounding joint instability (Figure 1A). Negative ulnar variance was also observed. For comparison, imaging of the right wrist was conducted, revealing no abnormal findings (Figure 1B). Additionally, a multiplanar multisequence magnetic resonance imaging (MRI) of the left wrist was conducted, revealing unremarkable findings in both the carpal and Guyon tunnels. The major ligaments of the hand appeared grossly intact. However, the osseous report revealed a diminutive appearance of the left lunate, showing signs of edema and sclerosis consistent with avascular necrosis (AVN). Furthermore, no fragmentation of the left lunate was noted. Nonetheless, bone marrow edema was present along the ventral lip of the left distal radius, which is believed to be degenerative in nature. Subsequently, subchondral marrow edema was noted at the scaphotrapeziotrapezoid joint, and ulnar minus variance was observed in the current alignment (Figure 2).

Ultimately, the MRI findings suggested changes compatible with KD, as well as the presence of a large radiocarpal joint effusion. The diagnosis and all treatment options for KD were thoroughly discussed with the patient. Among the options presented, the patient expressed interest in a radial shortening osteotomy to alleviate pressure on the lunate and restore blood flow, with the potential to halt disease progression and improve wrist function.

The patient was fully informed about the surgical procedure, including its associated risks, potential complications, and the recovery process. After further discussion and consideration, the patient opted to proceed with a left distal radius shortening osteotomy as the preferred management approach for KD.

## Discussion

KD, also known as AVN or osteomalacia of the lunate, was first presented in literature in 1843 by French doctor Peste who noted the collapse of the lunate bone in his studies [2]. Years later, Austrian radiologist Robert Kienböck further described the condition through examination of radiographic findings in 16 patients with osteomalacia of the lunate. Kienböck hypothesized that the osteomalacia followed an incremental progression, starting with isolated lunate involvement and ultimately progressing to radiocarpal involvement with degenerative changes [2].

Currently, KD is thought to arise from a combination of circulatory, anatomic, and traumatic factors, which ultimately causes decreased lunate perfusion and ensuing osteonecrosis [2]. Today, KD remains a very rare condition with a poorly understood etiology. In one retrospective study including over 150,000 radiology reports, the prevalence of KD was identified in only 1 of 15,000 cases (0.0066%) [3]. Over the years, additional research has been done to characterize the etiology of KD. Research suggests that KD predominantly affects males, peaking between ages 20 and 40, especially in those with comorbidities such as type 1 diabetes, systemic lupus erythematosus, and Legg-Calvé-Perthes disease [4]. Further research has been conducted to elaborate on the etiology, including a systematic review of the literature comprising 152 articles, in which researchers identified the four most commonly cited factors of the “at-risk” patient: negative ulnar variance (as seen in our patient), primary arterial ischemia of the lunate, trauma, and hand-arm vibration [5]. The negative ulnar variance is thought to increase stress on the lunate by preventing the ulna from properly distributing the load, leading to greater pressure on the radiocarpal joint [6]. Regarding “hand-arm vibration,” it appears that certain behaviors, such as using percussive tools in demanding occupations like construction and mining, may predispose individuals to KD. This is hypothesized to be due to repetitive exposure to low-frequency vibrations that may cause microtrauma, leading to bone necrosis. Although not statistically significant in the meta-analysis, further research is needed to better understand the relationship between occupational behaviors and the development of KD [5]. Recent research also suggests that there may be a genetic component, as the risk of developing KD was elevated in first-degree relatives and significantly clustered in multigenerational pedigrees [7]. These findings suggest that certain individuals may be genetically predisposed to KD, particularly those with affected first-degree relatives or a positive family history.

While virtually all patients typically present with wrist pain, KD can present radiographically in several ways. KD is typically classified by the Lichtman classification, which divides osteonecrosis into four distinct stages (Table 1). The Lichtman classification system was first developed using radiographs, but over time, researchers began incorporating advanced imaging methods like MRI [8]. The system ranges from Stage I, with a normal or minimally changed lunate, to Stage IV, with advanced lunate collapse and generalized wrist arthrosis [8]. In this case, the findings are most consistent with Lichtman Stage II, characterized by lunate sclerosis without collapse. This is demonstrated by the increased density of the lunate on radiographs and the absence of carpal malalignment or signs of advanced degeneration on both MRI and radiographic imaging.

**Table 1:** Lichtman classification stages. Classification outlining the progressive stages of lunate deterioration. Adapted from Lichtman et al. [8]. MRI: magnetic resonance imaging.

Lichtman stage	Radiographic findings
I	Radiographically normal, presence of marrow changes or small fractures on MRI
II	Sclerosis of lunate without collapse
Illa	Lunate collapse with preserved carpal alignment and height without scaphoid rotation
Illb	Lunate collapse with carpal malalignment, decreased height, and fixed scaphoid rotation
IV	Severe lunate collapse with scaphoid rotation and radiocarpal or midcarpal degenerative arthritis

This classification system enables practitioners to select the appropriate treatment modality based on the severity of the disease. To date, there are a plethora of treatment modalities that exist on the market (Table 2) [9]. Although there is no current standard, current research suggests that treatment should be guided by the Lichtman classification [10]. Thus, for less severe cases of KD, minimally invasive procedures such as splinting and core decompression can be done, while cases of increasing complexity may require proximal row carpectomy or total wrist fusion [10]. Further research should be conducted to determine the optimal treatment modalities for each stage of KD.

**Table 2:** Treatment modalities for KD. Treatment modalities for KD, ranging from conservative management to surgical interventions. Adapted from Danoff et al. [10]. KD: Kienböck’s disease.

Treatment modality	Description	Goal
Splinting	Keeping the wrist immobilized	Manage symptoms and prevent damage
Core decompression	Creating small openings in the lunate	Improve blood circulation and prevent deterioration
Vascularized bone graft	Grafting bone with its original blood vessels	Bring fresh blood supply to the lunate
Radial shortening osteotomy	Shortening the radius bone	Relieve stress and prevent further damage
Radial wedge osteotomy	Adjusting the radius angle	Balance the wrist and decrease strain
Capitate shortening osteotomy	Reducing the size of the capitate bone	Decrease stress and slow disease progression
External fixation	Using an external device to hold the wrist	Provide stabilization during recovery
Pinning	Inserting pins to stabilize bones	Maintain alignment and prevent movement
Intercarpal arthrodesis	Fusing carpal bones together	Stabilize the joint and reduce pain
Lunate excision	Removing the lunate bone	Relieve pain and stop joint degeneration
Proximal row carpectomy	Removing the first row of wrist bones	Decrease pain while preserving some function
Total wrist fusion	Fusing the entire wrist joint	Provide stability and pain relief

## Conclusions

In summary, KD remains a rare and poorly understood condition characterized by lunate osteonecrosis. Its etiology is thought to be associated with anatomical, vascular, and traumatic factors, although new research suggests that behavioral risk factors and genetics may also play a role. Currently, diagnosis relies on clinical presentation and imaging, with proper staging directed by the Lichtman classification, which should ultimately serve as a guide for treatment selection. While a range of treatment modalities exists, from conservative management to surgical intervention, there is no universally accepted standard of care. Given the complexity and variability in disease presentation, further research is essential to refine treatment strategies and establish evidence-based guidelines for optimal patient outcomes.
